# Influences on Emergency Clinician Use of Health Information Exchange: Interview Study

**DOI:** 10.2196/75865

**Published:** 2025-10-20

**Authors:** Brian E Dixon, Umesh Ghimire, Benjamin Richter, Corinne Bowditch, Saurabh Rahurkar, John T Finnell, Joshua R Vest

**Affiliations:** 1 Department of Health Policy & Management Fairbanks School of Public Health Indiana University Indianapolis Indianapolis, IN United States; 2 Center for Biomedical Informatics Regenstrief Institute Indianapolis, IN United States; 3 School of Medicine Indiana University Indianapolis, IN United States; 4 Department of Internal Medicine Thomas Jefferson University Philadelphia, PA United States; 5 Department of Biomedical Informatics College of Medicine The Ohio State University Columbus United States

**Keywords:** health information exchange, medical informatics applications, qualitative research, information systems, information seeking, information needs, viewpoints on and experiences with digital technologies in health, emergency medicine

## Abstract

**Background:**

Health information exchange (HIE) supports clinical decision-making in emergency medicine settings. Despite evidence and policies that encourage the adoption of HIE, use by clinicians is limited. Moreover, few studies examine HIE use years after adoption by hospitals or clinics.

**Objective:**

This study aims to examine the perceptions and use of a mature, operational HIE system by emergency department clinicians years after its implementation.

**Methods:**

We interviewed 21 clinicians in various roles (eg, attending physician and nurse practitioner) across multiple health systems that participate in a statewide HIE network. We asked questions about their use of the HIE system and the factors that facilitate or inhibit use. Analysis of interview transcripts was guided by a theoretical framework derived from information systems theories describing individual perception of, and use behavior toward, HIE systems.

**Results:**

A total of 26 factors across 6 domains were identified by respondents. All respondents recognized the value of HIE for medical decision-making in the emergency department, and access to information via the HIE system was preferred over traditional methods of telephoning other facilities or waiting for faxed records. Ease of use, particularly single sign-on functionality, was recognized as a key facilitator of routine use, enabling clinicians to access a patient’s HIE record with a single click from within their electronic health record system. Access to integrated data and advanced search features supported clinical decision-making. Limited training and poor system usability were identified as barriers to use.

**Conclusions:**

Achieving widespread adoption and use of HIE systems globally will require a focused effort to address multiple individual perception and behavioral factors. Researchers, leaders of HIE organizations, and policymakers alike should leverage these factors to achieve the goals of HIE and interoperability.

## Introduction

### Background

The adoption and use of health information exchange (HIE)—the electronic transfer of patient-level data or information across diverse and often competing health-related organizations [1]—offers a number of benefits, including reductions in inpatient readmissions, reduced duplicate laboratory and imaging studies, and decreased inpatient length of stay [2]. Multiple studies have demonstrated benefits especially to emergency department (ED) care [3-8]. Moreover, HIE facilitates intelligent communication, including clinical decision support [9], remote monitoring [2], and automated data integration [10]. As HIE is linked to better outcomes and efficiencies in care delivery processes, US health policies, including the Health Information Technology for Economic and Clinical Health Act [11] and the 21st Century Cures Act [12], have systematically encouraged adoption and use across myriad clinical settings [13].

As a result of multiple federal- and state-level policies, HIE availability—as well as the types of HIE systems available (eg, community based and vendor based)—has increased in many communities across the United States [13,14]. In 2021, according to the Office of the National Coordinator for Health Information Technology (ONC), 61% of hospitals reported using an HIE system to electronically query or find patient health information from external sources, suggesting that most clinicians use HIE routinely in care delivery [15]. Moreover, the ONC reported that in 2021, four out of 10 hospitals participated in more than one type of HIE, and most of these hospitals reported exchanging data with a community-based HIE network [15].

However, despite clear policy incentives, robust literature demonstrating impact, increasing organizational adoption, and evidence of more widespread availability, the actual state of individual HIE use in clinical practice seems to be limited; for example, using log data from a community-based HIE in operation for >15 years, we observed low rates of use among health care providers across inpatient, emergency, and outpatient settings despite overall increased adoption and use over time [16]. Other studies and reviews have reported relatively low use by clinicians [17]. As a further complication, much of the existing evidence on clinician use and perceptions was generated when HIE was a novel intervention or in the period shortly after implementation. These studies documented multiple barriers to implementation and use, including HIE system conflicts with workflow as well as usability challenges [18]. Clinicians may also be frustrated by the lack of information available (eg, in terms of breadth and depth) from smaller-scale exchange efforts [19]. While these earlier studies are critical, HIE has since evolved, electronic health record (EHR) systems and workflows have continued to change, and modern HIE is no longer an innovation but rather a routine capability.

A better understanding of actual use would support system improvements and identify additional, and potentially valuable, use cases. This is particularly true in the ED setting, where clinicians may not have a longitudinal relationship with patients, and access to prior information may be difficult [20]. Prior research involving ED clinicians before the implementation of technologies for accessing past medical history found that these clinicians desired better access to prior medical information [19,20]. Despite the value of HIE in the ED, reviews suggest that the ED is becoming a less-studied setting [21]. This study sought to elucidate ED users’ perceptions and use of a mature and robust HIE system.

### Purpose

As knowledge of HIE use by health care providers is limited, we conducted a qualitative study of ED clinicians to capture their perceptions and use of an operational HIE system many years after its introduction in the community. Our goal was to better understand when HIE is useful and the context of HIE use in ED care. We hypothesized that clinicians would perceive HIE to be useful and effective; yet, HIE would not be used for every patient, given the availability of information from a broad array of sources, including the patient, family, and intraorganizational EHR data.

## Methods

### Study Design and Setting

This study used qualitative methods to explore the antecedents of HIE use factors that act as facilitators or barriers to its adoption within ED settings. We recruited ED clinicians with access to the Indiana Network for Patient Care (INPC), a community-based HIE network operational since the late 1990s [22]. The INPC is managed by the Indiana Health Information Exchange, which has systematically expanded the service area and capabilities of the INPC, integrating longitudinal data from 38 health systems that encompass >100 hospitals with EDs [23,24]. The INPC is routinely used as a platform for research [25], including innovations in HIE [26-29] and interoperability [30,31].

**Figure 1:**
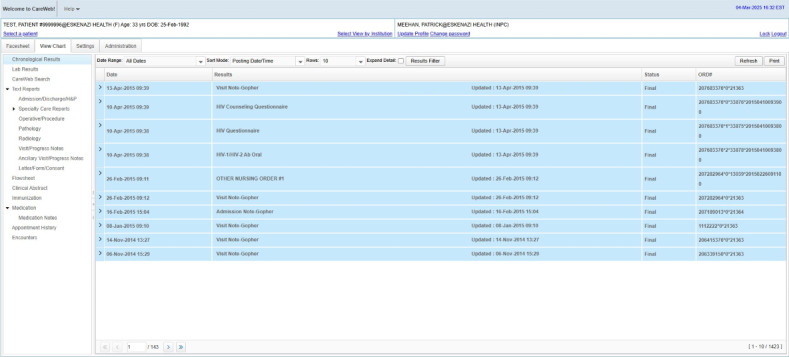
Screenshot of the CareWeb application that clinicians use to access patient medical records available in the Indiana Network for Patient Care.

Clinicians with access to the INPC interact with patient medical records via a web-based intelligent communication system known as CareWeb. Originally, CareWeb was developed using Java. The current version was redesigned in 2009 using ZK, an open-source Ajax web application framework written in Java for mobile and web apps. Users are managed by the Indiana Health Information Exchange, and updated user lists are provided regularly by each INPC organization. As depicted in Figure 1, the CareWeb system provides access to patients’ integrated, longitudinal medical records. By default, recent notes and results are displayed in reverse chronological order. The left-hand menu provides access to specific types of information (eg, immunizations, radiology, and discharge summaries) as well as CareWeb Search, an intelligent search that enables clinicians to query patients’ medical records. In the past, clinicians logged into CareWeb using an internet browser—outside of their EHR system (Multimedia Appendix 1). Currently, clinicians access CareWeb primarily via a button in their EHR system that launches a web browser and opens the same patient record that they have open in the EHR.

Our target participants were ED clinicians practicing in central Indiana, a region with the longest-established use of the INPC that includes urban, suburban, and rural communities. We purposely sampled clinicians with a goal of 30 users, stratified by clinical role (eg, physician, nurse practitioner or physician assistant, and registered nurse). The target sample was further stratified by level of HIE use, observed from INPC log files that measure the number of log-ins to CareWeb. We purposely sought to include low users (eg, approximately 1-2 log-ins per mo or up to 26 log-ins per y), medium users (eg, approximately 1 log-in per wk or 27-51 log-ins per y), and high users (eg, ≥52 log-ins per y, representing an average of ≥1 log-in per wk). We recruited clinicians across these strata until thematic saturation was achieved after 21 interviews [32,33].

### Theoretical Framework

**Figure 2:**
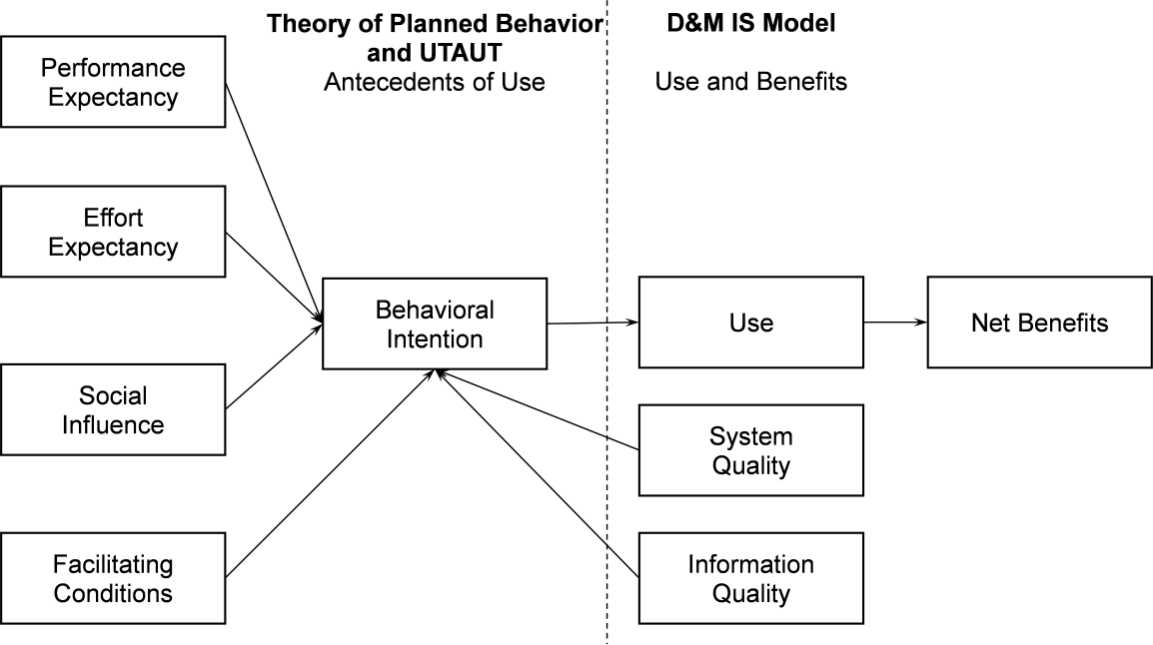
Theoretical framework used to guide the research. The framework integrates concepts from the theory of planned behavior, the unified theory of acceptance and use of technology (UTAUT), and the DeLone and McLean information systems (D&M IS) success model.

Our research is grounded in a novel integration of 3 conceptual frameworks from the information sciences (Figure 2). These frameworks are compatible and complementary, given that each theory centers on the concept of information systems (IS) use and its antecedents. The combined model allows for an examination of the factors that influence behavioral intention (BI) and use, which in turn influence net benefits. In the context of HIE use within ED settings, which manage a substantial proportion of clinical conditions, the integrated theoretical model facilitates a comprehensive exploration of the factors influencing ED clinicians’ intentions to use HIE as part of patient care. This exploration extends to understanding how these factors influence decision-making processes regarding individual care delivery and impact patient care outcomes. We hypothesized that a variety of factors, including system quality (eg, ease of use) and information quality (eg, data available in the INPC for patient care), would influence clinicians’ engagement with the HIE platform.

The theory of planned behavior posits that human behavior follows BI, which is influenced by a number of factors, including subjective norms [34]. This theory has been widely used in many fields, including health care, to explain behaviors as well as the factors that influence behaviors, such as vaccination uptake [35] or the adoption of EHR systems [36,37].

The unified theory of acceptance and use of technology (UTAUT) is a model that enables the measurement and study of the acceptance and use of technology and has been used to examine the adoption and use of EHR systems [38-40]. The UTAUT identifies 3 primary constructs that influence BI: performance expectancy, effort expectancy, and social influence [38,41]. Performance expectancy refers to the degree to which an individual believes that using the system will improve their performance. Effort expectancy denotes the perceived ease of using the system. Social influence refers to the degree to which an individual perceives that important others (eg, a supervisor or an influential colleague) believe that they should use the new system. The UTAUT posits an additional construct—facilitating conditions—defined as the individual’s belief that sufficient organizational and technical infrastructure is available to support the use of the system.

The DeLone and McLean IS success model is a multidimensional, interrelated framework for conceptualizing IS success [42,43]. The model involves 3 distinct but interrelated levels of success: technical, referring to system quality (eg, does the system perform as intended?); semantic, referring to information quality (eg, does the information convey the intended meaning?); and effectiveness, referring to the impact of the IS on the receiver (eg, user behavior, individual impact, and organizational impact). Success is defined in terms of net benefits to the user or the organization. The choice of “net benefits” is purposeful because there are always costs to implementing and using an IS. Furthermore, the model allows for an examination of the complex interrelationships between use and impact (eg, system use influences the products that in turn influence the individual user, which may in turn impact organizational outputs).

### Data Collection

Semistructured interviews with ED providers were conducted from February 2019 through January 2020 via Zoom (Zoom Video Communications, Inc), Skype (Microsoft Corporation), or telephone. Several respondents preferred to participate from home on their day off. Each interview lasted approximately 45 minutes.

During each interview, one researcher facilitated discussion while another took detailed notes. The questions aimed to elicit clinicians’ experiences of seeking information on patients from outside facilities; awareness and use of the INPC during ED encounters; perceptions of INPC tools, including the interface; and use of any other HIE tools, such as Care Everywhere. Probing questions were asked to dig deeper into clinicians’ experiences with HIE, particularly when responses were vague or involved specific use cases. All interviews were audio recorded and transcribed verbatim. The interview guide is presented in Multimedia Appendix 2.

### Data Analysis

We analyzed the interviews using a deductive approach. The initial codebook was developed by BED based on the theoretical framework and iteratively refined with input from the broader study team, including CB. Two coders (BR and MF) then analyzed the transcripts independently, meeting weekly throughout the process to review each other’s coding, discuss and refine codes and subcodes, and draw codes into broader themes. Any disagreements between the coders were resolved through discussion with BED. UG later independently reviewed and summarized these codes to ensure accuracy. Data were coded and analyzed using NVivo 12 (Lumivero) [44].

### Ethical Considerations

This study was approved by the institutional review board of Indiana University (protocol number 1710653338). All interviews were anonymized to protect participant confidentiality. Participants received a $50 gift card as remuneration.

## Results

### Participant Characteristics

We interviewed 21 ED clinicians drawn from 7 distinct health systems (Table 1). Of these 21 clinicians, 11 (52%) were physicians, 5 (24%) were registered nurses, 3 (14%) were nurse practitioners, and 2 (10%) were physician assistants. Most of the respondents (14/21, 67%) reported being in practice for 5 years, while 7 (33%) of the 21 respondents reported being in practice for ≤2 years (average 11, SD 8 y; range: 1-23 y). Nearly one-third of the respondents (6/21, 29%) reported dedicating between 80% and 100% of their work time to clinical activities, while others balanced patient care and administrative roles such as division chief, medical director, or manager. Participants were drawn from both large health systems (11/21, 52%) and community hospitals (10/21, 48%).

**Table 1:** Participants by clinical role and site (N=21).

Participant role	Large health system (n=11), n (%)	Community hospital (n=10), n (%)
Physician	6 (55)	5 (50)
Registered nurse	1 (9)	4 (40)
Nurse practitioner	2 (18)	1 (10)
Physician assistant	2 (18)	0 (0)

### Factors That Influence HIE Use

#### Overview

As summarized in Table 2, respondents identified an array of factors that influence their HIE use. A more complete synthesis of responses and example quotes from respondents is presented in Multimedia Appendix 1. In this subsection, we summarize some of the major themes from clinicians relevant to their use of HIE.

**Table 2:** Key factors, summarized by theoretical framework domain, that influence the use of health information exchange (HIE) by clinicians in emergency department (ED) settings.

Domains and factors		Illustrative quote
**Performance expectancy**		
	Changes to decision-making	“I was able to pull up their imaging studies from a week ago which helped guide my decision.” [R7; y in practice: 10]
	Continuity of care	“The ability to see that they were just at the hospital twelve hours ago...keeps us from doing unnecessary tests.” [R16; y in practice: 21]
	Frequency of use	“I probably use it every day for a few of my patients to confirm what happened at other hospitals.” [R5; y in practice: 15]
	Information retrieval in workflow	“Right after the patient left, I went back to CareWeb to double-check and then it changed the approach we were taking.” [R4; y in practice: 23]
	Patient characterization	“By pulling in all their previous admissions, I had a clearer picture of their frequent ED visits and issues.” [R9; y in practice: 7]
	Efficient retrieval	“It takes a few minutes to get through all the records, but it’s worth it when you’re avoiding duplication.” [R6; y in practice: 11]
	Motivation for information retrieval	“Patients come from all over, and to give them the best care, we need to know what’s been done at other hospitals.” [R10; y in practice: 5]
**Effort expectancy**		
	Desired features for ease of use	“I just think if I could make it so that I can stay logged in longer. So not constantly having to log in and log out.” [R2; y in practice: 14]
	Ease of use	“CareWeb is fairly self-explanatory, especially for information gathering rather than charting or placing orders.” [R1; y in practice: 21]
	Effort to retrieve information	“It took us a few minutes just to kind of read through, and they were all fairly much the same story.” [R1; y in practice: 23]
	Features that facilitate ease of use	“I like Care[Web]. I can often go into documents and copy and paste data directly into my notes.” [R2; y in practice: 14]
	Information display	“I think it may be organizing it differently...you can primarily organize by dates or something with tabs for labs, et cetera.” [R3; y in practice: 1]
	Speed of information retrieval	“It’s fast compared to a lot of our systems.” [R21; y in practice: 22]
**Social influence**		
	Discussion of use	“I first learned about using CareWeb when my colleague mentioned it as a helpful tool during our shift.” [R12; y in practice: 2]
	Expectation of use	“It’s pretty much expected now that we check the HIE before making any big decisions.” [R14; y in practice: 4]
	Resident use	“We always ask the residents to check CareWeb as part of their evaluation process.” [R11; y in practice: 13]
**Facilitating conditions**		
	Organizational support	“[A] patient came in saying they were just seen and had a test done...I asked the provider if I should contact the facility.” [R17; y in practice: 2]
	Team support	“Whenever someone has trouble accessing information, we work together to figure it out.” [R19; y in practice: 1]
	Training	“[A] colleague mentioned it as being useful. So that's kind of how I found out about it just working with coworkers.” [R4; y in practice: 23]
	Technical support	“We always reach out to IT if the system is slow or down, and they respond quickly.” [R13; y in practice: 9]
**Information quality**		
	Desired information	“I need to make sure that they haven’t had seven CT scans already in 2020.” [R2; y in practice: 14]
	Missing information	“I’ve had this before where a patient was recently discharged but the actual discharge summary is not in the system yet.” [R8; y in practice: 13]
	Missing institutions	“The VA has no records online that I know of anywhere, so you always have to call and get records from there.” [R1; y in practice: 21]
	Usability of information	“As long as we can get to it, that’s super helpful.” [R17; y in practice: 2]
**System quality**		
	Network reliability	“[H]ospitals created that VPN connection, so I don’t have a problem with that anymore.” [R8; y in practice: 13]
	System reliability	“If that link works...it’s so much more useful and quicker.” [R1; y in practice: 23]

#### Performance Expectancy

All respondents indicated that they knew about CareWeb and used it at least sometimes to look up information. Respondents reported that, overall, CareWeb enhances the retrieval of information for clinical decision-making. First, the HIE system provided access within “five to ten minutes” (R15; y in practice: 13), which was an improvement over waiting “fifteen to twenty minutes” (R19; y in practice: 1) or “up to an hour” (R13; y in practice: 4) to retrieve information from outside facilities. Moreover, one physician stated as follows:

A lot of times patients seem to fall apart in the evenings or at night when there’s no office staff, and the on-call person doesn’t want to or doesn’t have the capabilities to fax or email me the patient information.

R12; y in practice: 2

Second, most respondents reported that accessing CareWeb fits well into clinical workflow. Respondents generally accessed CareWeb either before they went into the examination room to see the patient or right after examining the patient to “double-check” information they obtained from their conversation with the patient.

Clinicians further viewed accessing the HIE system as critical to making good clinical decisions, with most respondents reporting that information from CareWeb had changed a clinical decision. Providers noted as follows:

Looking at that information can change what needs to be done in the immediate emergent future.

R2; y in practice: 14

The ability to see that they were just at the hospital twelve hours ago across town and got whatever treatment plan was there...keeps us from doing unnecessary tests.

R16; y in practice: 21

Reduction in duplicate procedures and tests, especially imaging, was frequently cited by clinicians as a major reason for looking up information in the HIE system. Some also reported experiences where information in the HIE system likely saved their patient’s life:

Had I not known how big it [tumor] had been before, he might have been discharged, and that could have had a poor outcome for him.

R12; y in practice: 2

Respondents also reported instances where the HIE system is unlikely to impact their performance as clinicians and therefore cases in which they do not feel that they need to use it. First, for patients whose care is immediate in nature, where past history might not make a difference in the care provided, some respondents indicated that using the HIE system would not be fruitful. Second, some clinicians were less likely to use it if patients reported that their prior care was delivered in the same health system. In these cases, health care providers used only the EHR to look up prior medical records.

#### Effort Expectancy

Respondents identified several effort expectancy factors that influence their use of HIE. With respect to factors that encouraged use, respondents highlighted ease of use, speed of access, comprehensiveness of data, SSO integration with the EHR, search capabilities, and features that allow information to be copied and pasted into their local notes. Factors that discouraged use included feeling overwhelmed by the volume of data in the HIE system and the system frequently logging out users.

Several aspects of the HIE system facilitated ease of use and supported clinicians’ effort. Several respondents reported that the system is intuitive to use or “fairly self-explanatory” and requires minimal training for basic functions such as accessing patient information, which encourages them to use it during clinical encounters. A frequently cited feature was SSO, which allows clinicians to click a single button in their home EHR to easily bring up the same patient’s records in CareWeb. Multiple respondents mentioned SSO as a key effort expectancy factor that encouraged HIE use. Clinicians who mentioned SSO functionality were generally those who had practiced for several years and compared accessing the HIE currently to a previous time when accessing it required a separate username and password:

Being able to click the button in Cerner that opens and goes directly to the patient’s chart without me having to remember and enter another password...has dramatically increased how often I use CareWeb compared to the way it was ten years ago.

R15; y in practice: 13

Respondents who did not report SSO as a feature were practicing in smaller community hospitals that had not yet implemented this feature, and these respondents often suggested that a more efficient log-in process would encourage their use of the HIE system.

Similarly, a few respondents reported that the search capability, which enables clinicians to conduct a Google-like search of the records in the HIE system, was a major enabling factor for wading through the comprehensive data available:

If you want to look up someone’s renal function or a CBC, you can just type in CBC in the box, and it’ll pull up the most recent ones. These little things are helpful to the clinician on the front lines.

R8; y in practice: 13

Respondents who did not mention the search feature reported that they did not know about it and that it would be a useful, desired feature:

Especially the lab results, it can get tedious clicking through [multiple pages].

R19; y in practice: 1

Finally, some clinicians reported that easily copying and pasting information from the HIE system into their notes within their EHR system was an important factor encouraging their use of CareWeb:

I can often go into documents and see if they’ve had a CT scan or MRI, then copy and paste that directly into my note. So, I find it the easiest to use.

R2; y in practice: 14

A few clinicians mentioned challenges with the volume and organization of data within the HIE system. While the respondents noted that comprehensive data were available to them, they felt overwhelmed by the volume of data and the fact that, by default, it is organized in reverse chronological order. These clinicians expressed a desire for tools that allow users to better sort and organize or filter the data to enable streamlined views that facilitate clinical decision-making. Similarly, some clinicians reported getting logged out of the HIE system when interrupted or during an encounter:

I just think if I could make it so that I can stay logged in longer. So not constantly having to log in and log out.

R2; y in practice: 14

Clinicians suggested that longer log-out times or ways to prevent involuntary log out would be an improvement. Such features would encourage clinicians’ use of the HIE system.

#### Social Influence

Respondents discussed several social influences on HIE use. Many respondents credited colleagues with introducing them to CareWeb:

A colleague mentioned it as being useful, so that’s how I found out about it.

R3; y in practice: 1

Most respondents who trained in Indiana were introduced to it during residency. A clinician who supervises trainees stated as follows:

I often have to prompt my learners to look in CareWeb.

R18; y in practice: 6

Other clinicians made the following observations:

The residents encourage us to use CareWeb more than we encourage them.

R19; y in practice: 1

We all use it. It’s extremely helpful.

R1; y in practice: 21

All these responses suggest that for most clinicians, at least those in training hospitals, there is broad use and a social contract to use it regularly.

Social influences were less clear in community hospital settings. A clinician practicing in a community hospital stated as follows:

I think some of [my colleagues] are still requesting records from the coordinator...not knowing what that kind of information exchange is.

R7; y in practice: 10

Therefore, social influence is not ubiquitous across Indiana despite the fact that the HIE system has existed for >25 years.

#### Facilitating Conditions

Health care providers discussed several organizational and technical factors that acted either as facilitators or barriers to use. Some respondents worked in organizations that actively promoted HIE use through experienced colleagues who encouraged their peers to use the system. A nurse practitioner stated as follows:

[A] patient came in saying they were just seen and had a test done...I asked the provider if I should contact the facility for records, and he showed me the CareWeb log-in.

R17; y in practice: 2

However, few respondents indicated that their organization embraced the HIE system. Rather, their colleagues showed them how to use it or encouraged their use:

It’s never been encouraged, and I’ve worked at three different hospitals here in [city].

R20; y in practice: 21

[I] don’t know if it’s a cost issue or utilization, um, but I think not having an auto enrollment, um, especially for providers is a disadvantage just because it’s not available.

R17; y in practice: 2

Therefore, organizational processes may not be set up to encourage HIE use.

A lack of organizational support for HIE was also evidenced in comments from multiple respondents who indicated that they learned how to access and use the HIE system from colleagues or on their own:

I just sort of started using it and figured it out myself.

R11; y in practice: 13

Another respondent stated as follows:

We don’t really include it in our formal onboarding or training of physicians.

R1: y in practice: 21

Moreover, none of the respondents indicated that the HIE system or anyone from the clinical organization provided updated, refresher training on the system:

There was never like, um, information session on how it can be used in a different, different ways that you can navigate through it that might be more beneficial.

R3; y in practice: 1

However, respondents did suggest that they perceived sufficient technical support to use the system when things did not seem to work:

I don’t feel as frustrated now because I know I can rely on 24/7 technical support—there’s always someone available.

R14; y in practice: 4

Other respondents similarly stated that they could contact the HIE organization directly or their hospital IT team and receive technical assistance with access or use.

#### Information Quality

All respondents emphasized the critical importance of information quality in the ED. The dimensions of quality mentioned often included completeness and information timeliness. The respondents expressed a strong desire for access to detailed past medical records, specifically laboratory results, imaging reports, encounter notes, and discharge summaries:

I need to make sure that they haven’t had seven CT scans already in 2020.

R2; y in practice: 14

Many respondents noted that the availability of timely information, such as recent diagnostic tests, was critical for swift decision-making in the ED.

Although most respondents said that the INPC generally possessed needed information, they also identified gaps. Some respondents reported incomplete access to records from specialized care settings, such as psychiatric facilities or smaller independent health care providers:

Most patients get cardiology care at XYZ, and I almost never find an EKG from them.

R11; y in practice: 13

A frequently mentioned institution was the Veterans Health Administration, from which clinicians strongly desired information on encounters but perceived it as obtainable only after first making a telephone call to request details by fax. In addition, respondents highlighted difficulty in accessing raw imaging data, with some relying solely on reports due to the time constraints inherent in ED workflows.

#### System Quality

Nearly all respondents indicated that the INPC was reliable, especially when accessed through the virtual private network (VPN) connection that most were using for their EHR system. Few clinicians reported technical challenges, apart from a provider who had difficulty with their VPN connection from home. An experienced provider who has accessed the INPC in multiple ways over the years noted that VPNs and other modern networking solutions have reduced technical barriers to accessing the INPC, thus motivating increased HIE use. System upgrades and downtime, when they occurred, were noted by clinicians as challenging. However, most respondents noted that these events occurred infrequently and were no more bothersome than when their EHR systems experienced downtime.

### Summary of Findings

Most ED clinicians had practiced in Indiana for >10 years (11/21, 52%) and reported being frequent users of the Indiana HIE, although several were categorized as low users based on system log-in events (eg, approximately 1-2 log-ins per mo). Knowledge of HIE was generally acquired during residency or through informal peer support on the job. Some clinicians received information about the CareWeb system when joining Indiana-based health systems from out of state. Clinicians unanimously emphasized the critical role of CareWeb in accessing patient information for informed decision-making and improving continuity of care.

Most clinicians recognized the utility of the HIE system while identifying areas for improvement, particularly in accessing the most relevant subset of information for clinical decision-making. HIE use was constrained by data availability, with most clinicians estimating its use for approximately 20% of their patient population. Ease of use, particularly the SSO functionality, was recognized as a key facilitator, allowing access with a single click from the EHR system directly into the patient’s CareWeb record. Although clinicians noted the usability of CareWeb for retrieving data from multiple institutions where their patients received care, they identified gaps in institutional participation within the HIE network.

The clinicians reported that the system was reliable and fast, and they appreciated the availability of support for troubleshooting when needed. Nevertheless, many were unfamiliar with advanced features such as the intelligent search function, which could enhance efficiency by streamlining information retrieval compared to manually scrolling through multiple encounter records. Persistent gaps in training and limitations in system usability were identified as significant barriers to HIE use.

## Discussion

### Principal Findings

This study of ED clinicians working in various health systems with access to a mature, statewide intelligent communications system highlights the complex, interrelated, and multifaceted factors influencing HIE use. Although purposely sampled to include clinicians who rarely used the HIE system and those who use it regularly (based on log data), respondents generally reported using CareWeb regularly. The HIE system was overwhelmingly viewed favorably by the clinicians, who unanimously preferred accessing information from other hospitals via the system rather than relying on telephone calls or waiting for faxed records. Multiple effort, performance, social, and organizational factors encouraged HIE use. These included the level of integration with their EHR (eg, SSO and an “easy button”), access to comprehensive information that influenced decision-making, and peers who encouraged and demonstrated HIE use. Nevertheless, several clinicians reported multiple effort, performance, and organizational factors that discouraged use. These included information missing from specific sources, information overload, and lack of training. The findings have implications for the advancement and adoption of HIE networks, including policies to encourage HIE use and strategies to enhance HIE operations.

### Importance of Research on HIE Use and Study Contributions

This study makes several important contributions to existing literature on HIE. First, the study used theoretical frameworks from the information sciences. A recent scoping review by Lum et al [17] identified gaps in HIE implementation studies and specifically encouraged researchers to “employ theoretical frameworks.” Second, most existing HIE literature focuses on organizational adoption of HIE systems and technologies (eg, whether a hospital reports having the capability to exchange information with outside hospitals [45,46]). Other studies have surveyed or interviewed clinicians before or just after HIE implementation [47,48], with virtually no research examining clinician perceptions years after implementation [19]. Moreover, few studies have assessed the level of use by clinicians, and while the annual American Hospital Association IT Survey asks organizations to report how “frequently do providers” use health information received electronically “from outside providers or sources,” it is unclear how hospitals distinguish responses such as “often,” “sometimes,” or “rarely” [49]. In this study, respondents characterized their frequency of use, with most reporting regular use and estimating HIE use for approximately 20% of their patient population. Third, respondent comments provide valuable context to prior work that examined the benefits of HIE [2,50], underscoring that data available through HIE networks indeed influence clinical decision-making, have benefited patient outcomes, and are perceived as highly valuable to clinicians. In other words, HIE systems are critical components of intelligent communications in health care, as evidenced by respondents reporting that HIE information enhanced their diagnostic reasoning, improved continuity of care, and led to a reduction in duplicate procedures and tests, often influencing clinical decisions and potentially improving patient outcomes.

### Implications for Advancing Adoption and Use of HIE

Interoperability and HIE remain key areas of focus for the Assistant Secretary of Technology and Policy and a global priority as specified in many nations’ digital health strategies. Achieving greater adoption and use of HIE will require attention to the factors described in this study; for example, existing studies suggest that hospitals with strong adoption of HIE functionality report higher levels of HIE use [51,52]. Therefore, many states and nations will need to continue to focus on performance expectancy factors, improving clinician perceptions of the value of HIE [46,53], enabling clinicians to apply HIE knowledge to clinical decision-making, and making the right thing to do the easy thing to do [54]. Approaches to support performance expectancy include asking clinicians to share why they use HIE, conducting quantitative assessments of HIE benefits, and performing mixed methods studies of clinician use cases. One example is a statewide survey of clinicians in Virginia [55]. Addressing challenges, such as the usability of HIE systems [56], seamless log-in mechanisms [57], and other effort expectancy factors, will also be critical to securing routine adoption and use. Seamless integration with EHR systems, search functionalities, and ways to optimize interaction with comprehensive patient data are additional effort expectancy strategies implied by this study to support clinician use.

Social and organizational factors are also critical to HIE adoption and use. A weakness of the INPC is the lack of training for newly hired clinicians. None of the respondents could recall learning about the HIE during onboarding or training by staff from the HIE organization. While training is often part of HIE implementation, ongoing training for both new and existing users years after implementation is important to sustain and expand use. HIE networks should help health systems incorporate training into onboarding of new clinicians, or the HIE organization should offer regular in situ training opportunities for new employees. Online, on-demand training might also be a strategy to deliver information to existing users about new features (eg, search functionality) or new data sources added to the HIE. Moreover, HIE networks should work with their health care facilities to establish a culture in which regular HIE use is expected, fostering resiliency that ensures use well beyond the implementation phase.

Finally, the findings from this study call into question existing metrics [58] of clinician HIE use. Respondents reported using the HIE system for between 5% and 30% of their patients. These data might help refine expectations around what constitutes frequent versus “sometimes” use. Moreover, they imply that there is a theoretical maximum proportion of patients for whom accessing the HIE yields value. Given that this study focused solely on the ED setting, a ballpark figure of 20% of patients might not be appropriate for every HIE use case. Still, these data suggest that work is necessary to refine metrics for clinician use, which may be lower than some stakeholders expect or promote. Regardless of the proportion, it is clear that clinicians value having information from HIE networks for those patients whose care and outcomes can be meaningfully impacted.

### Limitations and Future Directions for HIE Research

This study has several limitations. First, the study involved perspectives from clinicians on a single HIE network that has been operational for many years, and the participants primarily worked in health systems with established EHR use. Therefore, the results may not generalize to all HIE networks despite our efforts to purposely sample clinicians in different roles and practicing at hospitals of varied sizes and in diverse locations. Second, respondents worked in the ED, and perspectives from other specialties may differ. Third, despite our best attempts to interview clinicians who do not use the HIE system regularly, all respondents indicated that they use it on a regular basis. This might bias the findings because clinicians who purposely avoid using or have never used the HIE system were not interviewed.

While this study provides meaningful insights into HIE use in the ED setting, more research is needed to measure HIE use in various clinical settings and establish better indices for clinician use. Longitudinal studies on HIE use in relation to clinical outcomes could more firmly establish the evidence base needed to guide policy and practice. Cost-effectiveness studies involving clinician use data from logs could better demonstrate the value and return on investment of HIE infrastructure. Future research should examine the role of emerging technologies in enhancing HIE functionality, such as artificial intelligence summaries of clinical documents or comprehensive records, and their impact on clinician use. In addition, future research could explore potential differences in HIE use and perceptions across clinical roles (eg, physicians, nurses, and advanced practice providers) using a larger sample size and a mixed methods analysis approach.

### Conclusions

This study of ED clinicians’ perspectives illuminated several factors that facilitate or inhibit HIE use. As policymakers and health systems develop strategies to implement nationwide HIE, noting and leveraging these factors could be useful in achieving the goals of universal HIE adoption and use. Supporting clinicians’ performance and effort through advanced HIE technologies will be critical for adoption and for impacting care outcomes and costs.

## Appendix

Multimedia Appendix 1 Screenshot of the CareWeb log-in page and complete qualitative results of interviews.

Multimedia Appendix 2 Interview guide.

## Abbreviations

BI: behavioral intention
ED: emergency department
EHR: electronic health record
HIE: health information exchange
INPC: Indiana Network for Patient Care
IS: information systems
ONC: Office of the National Coordinator for Health Information Technology
SSO: single sign-on
UTAUT: unified theory of acceptance and use of technology
VPN: virtual private network
