# Supplementary material for: Influences on Emergency Clinician Use of Health Information Exchange: Interview Study
Source: JMIR Med Inform. 2025 Oct 20;13:e75865. doi: 10.2196/75865 (PMC12583940; doi:10.2196/75865)
Supplement: Multimedia Appendix 1 [file medinform_v13i1e75865_app1.docx]

# Multimedia Appendix 1

Influences on Emergency Clinician Use of Health Information Exchange: Interview Study from the Indiana Network for Patient Care

doi: 10.2196/75865

## Supplementary Figure

Screenshot of the login page for CareWeb, the web-based system used by clinicians to access the INPC.


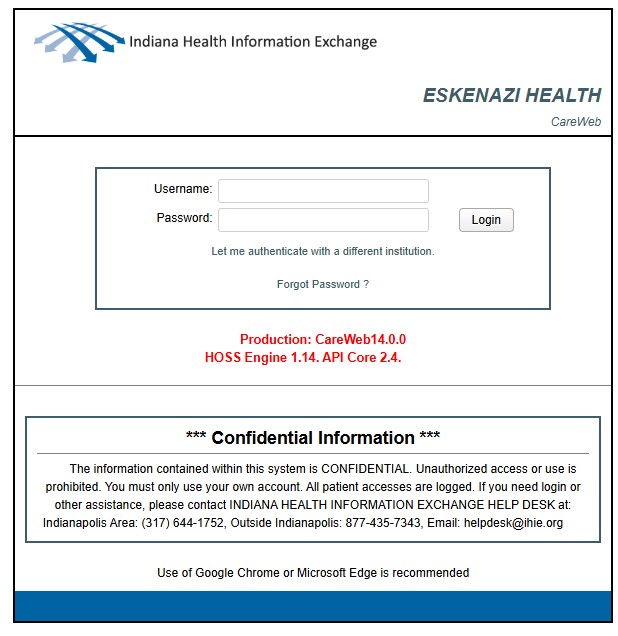


## Complete Qualitative Results of Interviews

### Domain: Performance Expectancy

#### Theme: Changes to Decision-Making

Providers reported that access to HIE data could substantially impact their clinical decision-making processes, particularly by preventing unnecessary repeat tests and guiding appropriate treatment plans. One provider highlighted the value of prior diagnostic workups reflecting the timesaving and cost-reducing benefits of HIE. A provider shared an example which portrays how reviewing previous assessments influenced their approach

*“Looking at that information can change what needs to be done in the immediate emergent future.” – (R2, 14y)*

*“If I hadn’t seen all the workups he’d already had done, I probably would have repeated a lot of them.” – (R1, 23y)*

Providers shared examples of incomplete or missing information within the HIE resulting in unnecessary procedures and overall suboptimal outcomes in absence of critical data.

“When the patient says, you know, I've had this procedure done… I can't look that up, I'm potentially doing more work on them.” – (R12, 2y)

*“Had I not known how big it [tumor] had been before, he might have been discharged, and that could have had a poor outcome for him.”- (R12, 2y)*

#### Theme: Continuity of Care

ED providers highlighted the importance of HIE systems, such as CareWeb, in enhancing continuity of care by providing seamless access and exchange of patient records across different facilities. Some respondents reported that previous records can avoid redundant interventions, streamline care, and align their treatment plans with those initiated at other facilities, thus optimizing patient outcomes.

*"The ability to see that they were just at the hospital twelve hours ago across town and got whatever treatment plan was there... keeps us from doing unnecessary tests and better focuses the care we provide." - (R16, 3y)*

Despite the perceived benefits, some ED providers expressed concerns about the potential pitfalls of relying on HIE systems. Respondents shared a cautionary experience stating an issue raised by was the incomplete or outdated nature of some records, which could lead to incorrect clinical decisions. Similarly, providers highlighted the frustration when crucial diagnostic reports, such as imaging results, were missing, leading to delays or the need for repeated tests.

*"It was nice to be able to read the resident's notes, but just that one key piece of information, knowing why they didn't put the description of the visit really would have framed my visit differently." - (R13, 4y)*

#### Theme: Frequency of Use

The convenience and accessibility of patient information significantly contribute to patient care usage, particularly when encountering patients new to their facility. Some providers highlighted how ease of access and integration into their workflow can motivate consistent HIE usage. Respondent 2 mentioned, "I probably do Care Everywhere first, just because that has an easier link for me."

HIEs become integral to their routine when managing high patient volumes or critical cases. Some providers find that HIEs like CareWeb are essential tools for delivering effective patient care, particularly in complex cases or when dealing with transferred patients.

*“I probably log into CareWeb and check those patients... probably 10 times a shift," - (R11, 4y)*

Some ED providers are less inclined to use HIEs due to inefficiencies or lack of necessity. HIEs are not always perceived as necessary, especially when the majority of the patient population remains within the same healthcare system. Furthermore, time constraints and the complexity of logging into multiple systems can discourage frequent use. A provider mentioned that the fast-paced nature of the ED environment often leaves little room for tasks, such as accessing external records.

*"I honestly don’t utilize that often—typically, we don’t look up information as ER nurses to be honest with you. We are so busy... I don’t think we have time to do that," – (R7, 1y)*

#### Theme: Information Retrieval

#### Sub-theme: Information retrieval using CareWeb, Cerner and Epic

Providers emphasized the ease of accessing critical patient information is important in ED settings. Providers noted that CareWeb, being integrated into the Cerner system, allowed them to seamlessly access patient records from other facilities, reducing the need for time-consuming processes such as faxing requests to external hospitals.

*"The first thing I do as soon as a patient checks in is review what we have in our EHR. If there’s not anything there, the next thing I do is click on CareWeb to see if there’s something from any of the other facilities." – (R15, 13y)*

*"It's great if they're on CareWeb because we can click straight from the Cerner medical record and bring up the CareWeb file and see what is there." – (R18, 6y)*

Providers frequently mentioned the ease of locating relevant documents, such as CT scans or MRI results, directly within their EPIC interface. In another example provider revealed the broad reach of EPIC in accessing patient records across various facilities, including out-of-state locations. The system’s ability to streamline documentation enhances workflow efficiency. These features of EPIC were seen as vital in improving patient care by providing timely access to comprehensive medical histories.

*"I like Care Everywhere. A lot of times I can just go into documents and see if they've had a CT scan or MRI and copy-paste that directly into my note." – (R20, 21y)*

*"Even if they go to a different hospital in our area, I can still pull through EPIC... I can easily get their information because of that." – (R13, 4y)*

Providers appreciated the embedded nature of these tools within the existing EHR systems eliminates the need for separate logins, further enhancing the efficiency and usability of HIE systems.

On contrary, providers expressed frustration when required information was not available within the HIE system or when navigating between different systems became cumbersome. CareWeb, while beneficial, was not always utilized due to the availability of information within the primary EHR system.

*"If they haven't been seen on the IU system, then you have to find a way to access that information... but I probably only need to use [CareWeb] about 20% of the time." – (R3, 1y)*

Additionally, the process of accessing CareWeb could still be seen as somewhat redundant, especially if initial searches within Cerner yielded sufficient data. Providers highlighted the potential for missed information if providers are not fully aware of the multiple avenues available within the HIE system.

*"Sometimes, if I had looked in a different place, I would have known that the Epic Everywhere button could see that." – (R12, 2y)*

Providers encountered obstacles with EPIC's Care Everywhere, particularly regarding incomplete or inconsistent data retrieval. While EPIC is a powerful tool, providers highlighted its limitations in providing detailed clinical information and suggested that its usability could be improved to better meet the needs of ED providers.

*"Sometimes Care Everywhere... pulls information, but sometimes it's just basically like a patient demographic face sheet." – (R14, 4y)*

*"I found that interface not as streamlined as some of the other ones." – (R8, 13y)*

#### Sub-theme: Information retrieval using fax and phone calls

Surprisingly, providers frequently rely on traditional methods like faxing or phone calls as a means of health information exchange. Fax was acknowledged as a reliable alternative when EPIC or other electronic means were not feasible.

*"Sometimes I'll have to get actual direct records from the facility. We'll call and have records faxed, particularly if they're not on CareWeb or Care Everywhere." – (R14, 4y)*

*"If I can't find the information in Cerner or CareWeb, then I have our coordinator start making the phone calls because oftentimes when I try to call the floor, you might call the facility, and then, you know, that department transfers you to medical records" – (R17, 2y)*

Providers gained the ability to obtain critical patient records through fax, particularly from facilities not participating in Care Everywhere. Some providers emphasized the importance of having a fallback method for information retrieval. One primary reason for reverting to traditional methods was the limited availability of information outside of regular business hours.

*"Um, so yeah, so sometimes I'll have to get actual direct records from the facility. So we'll call and have records faxed, particularly if they're not on CareWeb or Care Everywhere." – (R14, 4y)*

*"A lot of times patients seem to fall apart in the evenings or at night when there's no office staff, and the on-call person doesn't want to, quote, doesn't have the capabilities to fax or email me the patient information." – (R12, 2y)*

On the other hand, providers described the manual process of retrieving patient information via fax as cumbersome, primarily due to time delays and the dependency on external facility cooperation. Other providers echoed similar experiences, emphasizing the limitations of faxing as a less favorable option for timely information retrieval.

*"I got to call the other hospital, wait on hold... fill out the fax cover sheet, and then I have to fax the results to them." – (R6, 2y)*

The inefficiency of obtaining records by fax or phone calls during non-business hours is further compounded by the dependency on non-integrated systems.

*"It's a very time-consuming labor-intensive process usually to find the number and to get the person on the phone, and, um, oftentimes the- you know- it's at two A-M it's even harder" – (R20, 21y)*

#### Theme: Information retrieval in workflow

Many respondents agreed that CareWeb and Care Everywhere are valuable tools for consolidating information after initially assessing patient records, thus enhancing clinical efficiency and patient care. Providers noted that CareWeb, being integrated into the Cerner system, allowed them to seamlessly access patient records from other facilities The ease of accessing comprehensive records from multiple hospitals through a single interface encouraged them to use HIE.

*"It's beneficial to know if that's their baseline... in that situation, it's good to compare it to old records." – (R3, 1y)*

ED providers, on the other hand, also faced several discouraging factors when using HIE systems, which can limit their adoption. A significant barrier is the complexity and unfamiliarity of certain interfaces, such as Care Everywhere, which some providers find cumbersome compared to more familiar systems like CareWeb. Additionally, the need to navigate through multiple facilities' records individually within CareEverywhere, as opposed to the more streamlined presentation in CareWeb, can be time-consuming and frustrating.

*"I've been using CareWeb for over 10 years... it's just a system I'm more familiar with." – (R8, 13y)*

#### Theme: Patient Characterization

Many providers encountered scenarios where patients have recently been seen at other facilities but present to their ED without prior records. Providers find that when HIE systems are functional and accessible, they can streamline patient external records and avoid redundant testing. For example, if a patient presents with abdominal pain and has had a recent CT scan at another facility, the HIE system allows providers to retrieve and review these results promptly. The following examples highlight how the availability of external records can significantly affect the efficiency and accuracy of patient care:

*“A very common one would be somebody who was treated at another facility…That’s probably the most common that I see is when a patient was recently seen or treated at another facility.” – (R8, 13y)*

*“If a patient presents with chest pain, but I work in basically all the sites that I work at patients oftentimes get their care in other larger facilities…we usually have to do some digging to try to get those previous records.” – (R11, 4y)*

Providers have expressed disappointment with critical information from other facilities is either inaccessible or challenging to retrieve. This difficulty is compounded when providers are unaware of the existence of relevant records, making it less likely that they will seek out or utilize the HIE system. Moreover, some have highlighted concerns regarding the reliability of record access.

*“One of our sites is on the ethernet. So, it's not infrequent that we'll get patients that are just traveling through…we would have to call the other facility to have them fax records over.” – (R14, 4y)*

*“I find that to be a lot of times with St. Vincent they don't have a lot of their information on care web. So that does become an issue because we do see a lot of cardiac patients and who get their cardiac care at that facility and we don't have access to it.” – (R14, 4y)*

*“If I don’t know that there’s going to be something in a hospital, especially if I know roughly what kinds of tests they got, then I check. I have the secretary call and try to get their records.” – (R19, 1y)*

#### Theme: Efficient Retrieval

Providers reported that utilizing HIE systems such as such as CareWeb, significantly reduces the time needed to obtain patient information from external facilities. They noted that CareWeb's capability to rapidly retrieve critical data, such as recent test results and discharge summaries, is instrumental in facilitating prompt and informed clinical decisions.

*“If I can use CareWeb, I usually find the information within five to ten minutes.” – (R9, 2y)*

*“I wouldn't say it takes more than five to ten minutes to find what I need in CareWeb. It's usually not too time-consuming.” - (R15, 13y)*

However, providers also faced challenges that rendered the process more time-consuming. Many expressed frustrations with delays in accessing information and noted that administrative tasks often overshadowed clinical activities.

*“If the system is down or the information is hard to find, it can take up to an hour to get what I need.” - (R13, 4y)*

*“It usually takes me about fifteen to twenty minutes if I have to call and request information from another hospital.” - (R19, 1y)*

#### *Theme: Motivation for Information Retrieval*

#### Sub-theme: Avoiding Care Redundancy

Most ED providers highlighted the value of HIE systems in preventing unnecessary diagnostic testing and redundant care. **Providers emphasized that access to a patient's recent medical history can lead to immediate adjustments in the treatment plan, ensuring efficient and effective care.** Respondent 1 mentioned, *"If I hadn’t seen all the workups he’d already had done, I probably would have repeated a lot of them."* They noted that HIE not only helps avoid redundant care but also improves patient outcomes and optimizes resource utilization within the healthcare system.

*"It makes a huge difference because patients now just go all over the place getting care, and so we can really minimize over-testing or inappropriate treatment by seeing what their past information was." - (R11, 4y)*

On the other hand, inconsistency and incompleteness of the data available through these systems were noted as barriers to effective HIE use. Providers expressed frustration when critical patient information was missing or not easily accessible, leading to delays or unnecessary repetition of tests. Additionally, the patchy availability of information across different healthcare systems often leaves providers operating with incomplete knowledge, which can compromise the quality of care.

*"If you can't find it right away and you're in the ER, you can sometimes just get frustrated and obtain another set of unnecessary imaging if you can't find it right away." - (R3, 1y)*

The lack of accessible records led to unnecessary patient transfers, further complicating the process.

*"If I had had access to the records, I totally would have avoided that transfer; it would have been probably a better disposition for the patient." - (R11, 4y)*

#### Sub-theme: Desire to Provide Good Quality of Care:

Providers primary motivation for utilizing HIE systems is driven by a deep-seated desire to deliver high-quality care. One of the enabling factors to make informed decisions, particularly when dealing with complex cases or when there is a need to verify if a patient's current condition deviates from their baseline health status, is the accessibility of prior patient information, such as lab results, imaging studies, and detailed medical histories. Providers expressed the importance of comparing current lab results with historical data to determine whether observed abnormalities are new or longstanding conditions.

*"As a treating ER physician, it may be helpful for me to look up and see, okay, what tests has this patient had done?......... they've seen consultants who that's going to help me treat them at that point in time when I'm seeing them." - (R8, 13y)*

**Despite the desire to provide the best quality care, providers expressed disappointment when the information they needed was either not present or incomplete, which could impede their ability to provide optimal care.** For example, one provider pointed out that when accessing imaging data, accompanying reports were missing, which is crucial for understanding the imaging data. This lack of comprehensive data diminishes the utility of HIE systems and discourages providers from relying on them.

*"We don’t get reports with them so and then they’re helpful to quickly like you don’t want to have to go through the [inaudible] or have radiology read it to be able to look through the report for basic information about what was in that scan." – (R4, 1y)*

**There is a sentiment of inequity among providers regarding information sharing.** Some providers are discouraged by the fact that while they are expected to contribute data to HIE systems, they do not always receive reciprocal access to patient information from other providers or institutions. Providers questioned why other physicians would withhold patient information when sharing it could significantly enhance patient care outcomes.

*"Because let's be honest, their physicians are utilizing the information we provide. So why aren't they providing us their information? You know, it's not like we're going to scout their patient from them. They're clearly an established patient. So it's all about patient care and giving the most optimal patient care. So, they're kind of impeding that." – (R14, 4y)*

#### Sub-theme: Drug-Seeking Behavior

Accessing comprehensive patient information through HIEs enables providers to deliver higher quality care by preventing the repetition of inappropriate or harmful treatments. Providers shared that HIEs allow them to identify and minimize harm when dealing with patients who have a history of opioid misuse by checking if the patient had received opioids from other hospitals. One provider emphasized that HIEs help verify the accuracy of a patient's reported medical history, particularly when the patient is not forthcoming about past treatments or drug use.

*"And also, with his drug problems, being able to identify his multiple requests for opioids and try to intervene on that or at least minimize harm...we could look and see which doctor had been taking care of him in order to try to facilitate a transfer for admission instead of trying to start from scratch." – (R11, 4y)*

*"We can see that trend, they should have this x amount of some already, but they are saying they don’t have any pain medication so you know, it's kind of a trend that you can see, and that’s what we see on our floor a lot of times." – (R7, 1y)*

Some providers noted that incomplete or outdated information in the HIE system can hinder their ability to make informed decisions. An ED provider described the limitations of HIEs, explaining that when data is not comprehensive, it can lead to potential gaps in patient care. Failure to locate a patient's past record in the statewide chart can delay the accurate diagnosis of a serious infection related to intravenous drug use.

*"I had a patient who I was convinced that there was something wrong with him...I had looked in the statewide chart and didn’t find anything...but when I was doing my chart a few days later, that epic connect actually showed that visit...he was seen for IV drug use." – (R12, 2y)*

Most providers shared concerns about the trustworthiness of the patient's story, particularly when the patient's narrative does not align with the information available in the HIE. In such cases, the provider must rely heavily on the HIE data to verify the patient's claims, which can be challenging if the data is insufficient or if the patient has a history of visiting multiple hospitals.

*"The other piece in the emergency department that I always try to look up is one that I am getting is occupied somewhere. I don't quite either trust their story or trust their medical history." – (R12, 2y)*

#### Sub-theme: Reviewing medical history

Providers are motivated to use HIE to access critical past medical records, especially when patients have received care at multiple healthcare systems. Providers shared their experience of accessing quick information that helped them review past medical data for informed decisions and improved patient outcomes. For example, one provider emphasized the importance of HIE in piecing together a patient's medical history.

*"When patients bounce between different hospital systems, digging into their records is essential to find everything that happened, especially if they’ve had significant testing or admissions elsewhere." – (R1, 23y)*

Some providers expressed that the quick retrieval of information was an advantage, particularly when patients may be confused or unable to provide accurate medical history.

*"It’s helpful for me to look up and see what tests they’ve had done, like imaging or procedures at other facilities, which helps me treat them better at that moment." – (R8, 13y)*

When sharing concerns about fishing for information, a common issue raised by many providers was the time and effort required to retrieve information from multiple sources. Providers stated that the process of retrieving information could be lengthy, especially when the system was slow or unresponsive. Additionally, respondents expressed concern over the variability in data availability across different HIE systems, combined with system inefficiencies, which could lead to frustration as providers may not always find the necessary information despite their efforts.

*"It’s pretty common for me to look on CareWeb and not find anything, which is frustrating, especially when I’m trying to confirm a significant part of the patient’s history." – (R19, 1y)*

*"In a busy ER, logging into CareWeb can sometimes be slower than just asking the patient directly, especially when the system doesn’t have all the records, and you’re left with incomplete information." – (R6, 2y)*

#### Sub-theme: Measure Outcomes

Several participants highlighted how HIE facilitates follow-up on patients transferred to other facilities, enabling providers to track patient outcomes and enhance their clinical skills. The benefit of using HIE to revisit cases after shifts was emphasized by the providers which extends the care quality beyond the immediate clinical encounter. Similarly, providers emphasized the ability to access information quickly after patient transfers as essential for providing timely feedback to staff.

*"I use it not only in real-time during patient care but also after the patient leaves for my own clinical improvement and to assist in teaching medics." – (R8, 13y)*

*"I like to do my follow-up quickly because our staff likes to have that kind of feedback, like hey, we transferred this patient out...can you get me a follow-up?" – (R9, 2y)*

Contrarily, some ED providers voiced that there’s some limitations and inefficiencies regarding incomplete or delayed availability of patient records, which hinders the ability to perform timely follow-ups. Also, the variability in the quality and timeliness of information from different facilities was noted as a significant barrier.

*"I go in and try to look at their discharge summary...but lately, there have been a lot of documents missing. I can’t see them, not even a simple EKG." – (R10, 5y)*

*"Sometimes, if the facility has access, I can see [the patient’s clinical course], other times not...it can take weeks or months to come back to us." – (R13, 4y)*

#### Sub-theme: Verifying Patient Information

**Providers expressed that they often encounter situations where patients either inadvertently omit or deliberately withhold important medical history details, which can impede care.** Furthermore, providers emphasized that the use of HIE allows them to cross-check and verify patient-reported information against records from other facilities. Moreover, providers re-emphasized that having access to previous imaging results or lab records can significantly alter the management plan.

*"Sometimes patients, of course, are not always honest, and they don't always tell us they've been somewhere else. But we can actually look and see if they’ve been anywhere recently, which helps us deliver good patient care." – (R2, 14y)*

*"It may be helpful for me to look up and see, okay, what tests has this patient had done at other facilities? That’s going to help me treat them at that point in time when I’m seeing them." – (R8, 13y)*

One primary difficulty in verifying the patient information that providers perceived was the complexity and time-consuming nature of navigating these systems, which can disrupt workflow efficiency. The process of retrieving and interpreting data was frustrating if the data is incomplete or not presented in a user-friendly manner. This issue is exacerbated when the retrieved data does not align with the current patient situation, leading to confusion and potential delays in care.

*"Just because I don't have information on when it was done or they can tell me roughly when it was last time, but if it's not in [the system], I don't know what it shows." – (R12, 2y)*

Providers express demotivation as the legal and ethical concerns associated with relying on patient-reported information without verification from HIE records. This concern was highlighted - *"How much do you want to rely on the lay person for the new thing? My CAT scan showed this without an official report—that may not give them the best care and then that sets someone up for a potential lawsuit later on." – (R12, 2y)*

### Domain: Effort Expectancy

#### *Theme: Desired Features for Ease of Use*

Respondents discussed the ease with which users interact with the HIE system, highlighting their experiences with desired features within the system. Several respondents emphasized the need for more user-friendly and intuitive interfaces. They sought features that would streamline the process of accessing and utilizing information, reducing the cognitive effort required. For example, respondents expressed a preference for features such as single sign-on, SSO), better search functionality, and the ability to sort, filter information, and display relevant information. Respondent noted that these features reduce the cognitive load, allowing clinicians to quickly identify and locate the data they needed, saving time and focusing on patient care. Additionally, respondents suggested that CareWeb should integrate more seamlessly with other electronic health record systems, eliminating the need for manual data entry and reducing the likelihood of errors. This would simplify the workflow for providers and improve the overall efficiency of using CareWeb. However, when these features were absent or poorly designed, it often led to frustration and hindered system adoption. Suggestions included making text more searchable and improving the organization of different types of medical records.

*“I just think if I could make it, and maybe this is just me, I could make it so that I can stay logged in longer. So not constantly having to log in and log out. That would be helpful too.” – (R2, 14y)*

*"* *I think it may be just organizing it differently. You can primarily organize by dates or something, with different tabs for labs, radiology, progress notes, and previous diagnoses. And without having to click on the drop-down box to see those easily." – (R3 (1y)*

There is a strong interest in integrating CareWeb more seamlessly with other electronic health record systems, especially for those using iPads or other mobile platforms for simplified workflow, enhanced accessibility and efficiency.

#### *Theme: Ease of use*

Several respondents highlighted the convenience and ease with which clinicians could navigate the HIE systems. For example, one provider noted that the HIE system was "fairly self-explanatory" and required minimal training, mainly focusing on how to sign in and access patient information rather than more complex tasks such as charting or placing orders.

*“CareWeb is fairly self-explanatory, especially for information gathering rather than charting or placing orders. So there's not much training needed. It's really just about signing in and accessing patient information.” – (R1, 23y)*

Another provider emphasized the speed and availability of information retrieval as key benefits, describing the HIE system as "*really simple*".

The ability to quickly find specific information without the need to navigate through cumbersome processes was perceived as a substantial improvement over traditional method. Similarly, the centralization of information within a single system was appreciated for its efficiency, as it allowed providers to avoid the time-consuming task of searching through multiple platforms.

*“I think it's a convenience factor of being so available on your application. And the speed of it is really simple.” (R3, 1y)*

*“It's great because it's a central clearing house," making all necessary data "easily searchable" and "readily available." – (R14, 4y)*

Providers who had used the same system for an extended period expressed comfort and efficiency in navigating it, with one noting that they had been using the system for over ten years and found it *"easy to navigate and grab"* information. While respondents generally found CareWeb to be relatively easy to use, especially for those with experience using the system, they also identified areas for improvement. A common issue was the organization and accessibility of information within the system. For example, one provider expressed that HIE system was generally intuitive, however, the process of sifting through documents to find specific information was time-consuming.

*And it takes a lot of time to find the information you need. Like I said, there's not a lot of extra time to be looking through chart information. – (R3, 1y)*

Additionally, providers who had to work with multiple EMR systems found it difficult to remember the specific steps required to access certain types of information. The lack of uniformity between different systems posed a significant challenge. As one respondent explained, the differences in system organization sometimes required *"clicking in several different spots"* before finding the needed data, which could be frustrating and time-consuming. Furthermore, technical challenges also contributed to the difficulties in using HIE systems. Respondents reported that issues like slow loading times, problems with logging in and system functionality occasionally disrupted the workflow requiring intervention from IT personnel to resolve the issue.

*“Sometimes, it doesn't log you in correctly. You hit the button to return, but it doesn't log into CareWeb. Nobody really knows how to solve this problem, so it eventually gets reported to our IT people.” (R4, 1y)*

#### *Theme: Effort to Retrieve Information*

Most respondents generally found it straightforward to locate and access encounter notes, lab results, and other relevant data within CareWeb. Some providers acknowledged the benefits of HIEs, particularly in their capacity to consolidate information from multiple hospital systems into a "one-stop shop," reducing the need to visit individual sites. This consolidation is seen as a significant advantage, allowing for quicker access to a broader range of patient data. Additionally, the integration of cloud-based imaging has been highlighted as a positive development, although the lack of associated reports can limit its utility.

*It's pretty fast... and when the information is in the system, I'm usually able to find it pretty readily. – (R15, 13y)*

Some encountered challenges with the search functionality or the organization of information, which made it difficult to retrieve patient information. Providers expressed that the process of navigating multiple systems can be cumbersome and time-consuming. As one provider noted:

*“It might take me five to seven minutes to do that. You know, toggling through, copying and pasting, and synthesizing the information. Unfortunately, that's five to seven minutes lost from patient care.” – (R2, 14y)*

This effort-intensive process discourages some from fully utilizing HIEs, especially under time constraints typical in emergency settings. The availability and completeness of information within CareWeb could also vary depending on the provider who entered the data and the specific facility where the care was provided. Some respondents expressed a desire for a more simplified process for viewing trends over time and comparing results from different facilities. Another provider pointed out the challenge of sifting through extensive, sometimes irrelevant, data to find critical information, noting that even when data is accessible, the user interface can make it difficult to extract usable insights quickly.

*“And then you've got thousands of pages of stuff dumped in here. How can you make it useful? For example, you type in CBC, and you get the most recent ones, or if you want to look a year or two ago for comparison.” - (R8, 13y)*

#### Sub-theme: Encounter notes

Some providers expressed positive experiences when retrieving encounter notes, finding it straightforward to access past visit information. However, challenges were also highlighted, including the presence of extraneous information that cluttered essential data, making it difficult to locate relevant details quickly. Additionally, inconsistencies in document formatting across different institutions led to confusion, with critical information often buried under repetitive or non-essential content. This inconsistency and information overload impeded efficient retrieval, sometimes necessitating extensive scrolling through multiple pages to find the necessary details, particularly in emergency settings where time is critical.

*"It took us a few minutes just to kind of read through, and they were all fairly much the same story." - (R1, 23y)*

*"Sometimes I spend a lot of time scanning through multiple documents to try to find the meat of why somebody was there." - (R15, 13y)*

#### Sub-theme: Lab results

Providers reported both advantages and frustrations while retrieving lab results. Some providers appreciated the ability to find lab results when specific dates or tests were known, particularly when the data was well-organized by date. However, several issues complicated the process, such as needing to adjust date manually to view older results. The presence of excessive, non-essential data, like repeated normal values, further complicated searches, leading to tedious navigation through multiple pages to locate pertinent lab results. The inconsistency in how labs were organized across systems exacerbated these challenges, contributing to inefficiencies in patient care during emergency encounters.

*"You kind of have to switch the date range around... I kind of wish that everything would just pop up each time." - (R2, 14y)*

*"Especially the lab results, it can get tedious clicking through all of the pages... you have to click to like the fifth page." - (R19, 1y)*

#### Sub-theme: Patient lookup

The patient lookup feature allowed providers for quick access to patient information with minimal input, significantly streamlining the process. However, these benefits were not universal, as some providers experienced frequent logouts and difficulties with direct links, requiring repeated manual entry of patient details, which disrupted workflow. Some suggested the need for a more robust search function that could handle variations in patient names and demographic information. Additionally, respondents highlighted the importance of having accurate and up-to-date patient information, particularly in cases of common names or infants initially registered under their mother’s name, making it difficult to trace patient records across systems.

*"I have to log in every time, and it logs out pretty fast, so I pretty much have to log in every time between patients." - (R2, 14y)*

*"It automatically links up to the patients that you're looking at... it just kind of instantly pops up, which is kind of nice." - (R8, 13y)*

#### Theme: Features that Facilitate Ease of Use

#### Sub-theme: Copy Paste

Providers appreciated the efficiency of the copy and paste function in HIE, allowing them to seamlessly incorporate relevant patient data into their notes, which aids in continuity of care. However, there were some frustrations regarding inconsistent functionality across different systems while another highlighted the difficulty with certain systems. Overall, the ease of copying pertinent data directly into patient charts was seen as a significant advantage, although some systems' limitations posed challenges.

*" I like Care[Web]. I can often go into documents and see if they've had a CT scan or MRI, then copy and paste that directly into my note. So, I find it the easiest to use.” - (R2, 14y)*

#### Sub-theme: EHR Button

The presence of a dedicated EHR button that directly links to the HIE was viewed as a major facilitator of ease of use. Respondents noted that the convenience of a single-click access significantly reduced the time and effort required to retrieve patient information. For instance, one respondent described the process. Another provider appreciated how it eliminates the need for manual entry.

*"We actually have a click of a button, and it automatically launches us to IHIE with a single sign-on... it made it so much easier." - (R9, 2y)*

*"It automatically links up to the patients... so you don't have to input all their information." - (R5, 10y)*

Sub-theme: Search function
Respondents emphasized the importance of having a quick and accessible way to review a patient's social history, which can significantly impact medical decisions in emergency departments. Providers valued the ability to bypass irrelevant information and quickly locate the necessary records using the search function.

*If you want to look up someone's renal function or a CBC, you can just type in CBC in the box and it'll pull up the most recent ones. These little things are helpful to the clinician on the front lines.”- (R8, 13y)*

*It's easily searchable. If I need specific information, I can quickly search for it in one of the screens.”- (R14, 4y)*

This feature was particularly appreciated for its ability to focus on critical data, reducing the cognitive load on providers and improving the overall efficiency of patient care.

#### Sub-theme: Single Sign-On, SOS)

A key facilitating factor is the integration of SSO features within EHR reduces the cognitive burden and time spent accessing patient data, thus encouraging more frequent use. Respondents appreciated the convenience of SSO, which allowed them to access CareWeb without having to enter separate login credentials. Providers explained that SSO was particularly valuable for providers who used multiple systems within their healthcare organization.

*"Being able to click the button in Cerner that opens and goes directly to the patient’s chart without me having to remember and enter another password... has dramatically increased how often I use CareWeb compared to the way it was ten years ago." - (R15, 13y)*

*"It’s a single button. It’s not something I have to put in a username, and I don’t have to remember a password... that is very helpful." - (R17, 2y)*

#### Theme: Information display

Respondents expressed a desire for improvements in the way information is displayed within CareWeb. A common challenge noted was the cumbersome nature of navigating through disorganized information, requiring multiple steps to access relevant data, which could lead to frustration and inefficiency. They suggested that the interface could be more visually appealing and easier to navigate, with some suggesting that organizing information by specific tabs, such as labs or radiology, would significantly enhance usability. Additionally, respondents highlighted the need for clearer categorization of data and more prominent display of relevant information. This would help users quickly identify and locate the data they needed, reducing the cognitive effort required. Despite these challenges, providers also recognized the value of HIE systems in offering comprehensive patient histories, which aids in informed decision-making.

*“Everything’s all the text reports, which is what I usually go to, they’re all in some kind of chronological order with the date listed. Easy to see, so I can see like, which test was most recent." – (R10, 5y)*

*"The layout is a little bit more cumbersome. But that’s not to say it’s not fast and easy to use—it’s just a different layout that you kind of have to get used to." – (R3, 1y)*

#### Theme: Speed of Information Retrieval

Providers expressed that quick access to information significantly enhances efficiency and decision-making, which was seen as both a motivating and discouraging factor for using HIE. Providers highlighted the value of timely information in the ED. The ability to quickly retrieve recent medical records through HIE was emphasized as a significant advantage.

*“It’s fast compared to a lot of our systems.” (R21, 22y)*

*"Sometimes it’s kind of a struggle to live here, having to open one system, close it, then open the next one." - (R2, 14y)*

The need to navigate multiple systems and logins was referred to as a barrier to efficient use and can detract from the overall user experience and effectiveness. While HIE systems like CareWeb offer the advantage of quicker access compared to traditional methods such as faxing, they are often perceived as slow and cumbersome, particularly when providers need to access complete or specific patient information. One provider highlighted the time-consuming nature while navigating extensive records.

*"I know I've gotten frustrated and just walk away...and of course, I'm logged out when I come back in." - (R12, 2y)*

### Domain: Social Influence

#### *Theme: Discussion of Use*

Providers cited peer recommendation as a key motivator for using CareWeb. Providers often learn about the system’s utility from colleagues who share their positive experiences. Additionally, respondents expressed that the direct encouragement from experienced staff, especially during the training of new residents or students, reinforces their value of CareWeb.

*"A colleague mentioned it as being useful, so that's how I found out about it." - (R3, 1y)*

*"I’ll encourage [learners] to look into IHIE" - (R21, 22y)*

On the flip side, providers reported that discussions about CareWeb are infrequent, which may result in missed opportunities to leverage its benefits. Some expressed frustration when colleagues fail to utilize the system effectively. Providers raised concerns over gap in communication about the system’s advantages contributes to a variable uptake among providers.

*"I don’t know that I’ve ever really talked about it with my colleagues." – (R19, 1y)*

*"I often have to prompt my learners to look in CareWeb," – (R18, 6y)*

#### *Theme: Expectation of Use*

Providers consistently reported a positive reception from their peers regarding the use of HIE systems. Respondents agreed that the sharing of information through these systems is not only accepted but also highly valued, as it enhances collaborative decision-making among team members.

*“I think we all appreciate seeing what the other providers are thinking and seeing regarding patients.”– (R2, 14y)*

*This indicates that Another provider echoed this sentiment, stating, “We all use it. It’s extremely helpful.”– (R1, 23y)*

Despite this general approval, some providers expressed concerns about the time and effort required to effectively use these systems. They highlighted that issues such as efficiency and a lack of comprehensive understanding could hinder their utilization.

*“There’s a lot of pressure in the emergency departments should not delay the patient and keep things in a timely fashion…you can’t spend too much time navigating to your CareWeb.” – (R3, 1y)*

*“I think some of them are still requesting records from the coordinator…not knowing what that kind of information exchange is” – (R7, 1y)*

#### *Theme: Resident Use*

Respondents were generally more open to using the HIE, often viewing it as a valuable learning tool. They expressed that their use of the HIE system was influenced by their training and the attitudes of their supervising physicians. The integration of HIE systems into routine practices of medical residents significantly drives their usage.

*“The residents do utilize care lab and residency. So, they know how to use it” – (R11, 4y)*

*“I think the residents encourage us to use CareWeb more than we encourage them. Because they’re more used to it,” – (R19, 1y)*

### Facilitating Conditions

#### *Theme: Organizational support*

The assistance providers received from colleagues and managers in obtaining access to HIE systems was appreciated by the providers. Some providers reported that providers are often required to seek support from another organization when obtaining information from outside providers network, which often requires submitting a request. The willingness of organizations to support in this initiative was valued by the providers. For instance, one provider explained that after inquiring with their manager, they were guided through the process of submitting an online form to gain access to the CareWeb system. The perceived utility of HIE was also a motivating factor, with providers recognizing its potential value in patient care. As one interviewee stated, "Yeah, it'd be handy every once in awhile."- (R16, 3y)

*“...a patient came in saying they were just seen and had a test done...I asked the provider if I should contact the facility for records, and he showed me the CareWeb login.” – (R17, 2y)*

*"Um then the um CareWeb link um is- I think we had to independently sign up for it or request access and then it was added to our- um- it's a button on our screen." - (R17, 2y)*

Limited access was another significant barrier for HIE access providers mentioned. Many providers reported that their organizations did not actively promote or encourage HIE use, stating that as “*It’s never been encouraged, and I’ve worked at three different hospitals here in [city]*.” Moreover, the lack of auto-enrollment for providers into systems like CareWeb was seen as a major barrier.

*"Um, I don't know if it's really CareWeb or IU Health, but, um, I think you have to um submit a Solar request to have it added to Cerner." - (R17, 2y)*

*“… I don't know if it's a cost issue or utilization, um, but I think not having an auto enrollment um especially for providers is a disadvantage just because it's not available.”* - *(R17, 2y)*

#### *Theme: Team support*

Many providers reported that team support, including assistance from unit secretaries, coordinators, or charge nurses, was highly beneficial when delegating tasks such as contacting other facilities. Additionally, some providers noted that support staff with access to HIE systems could retrieve information on behalf of physicians, which was particularly helpful during busy periods.

*".. if the patients knows you know I came from whatever hospital, um they'll tell the physician who tells the secretary and then the secretary will start making phone calls to that facility." – (R9, 2y)*

*"Um even though all of our secretary's um they would usually be the ones to log in and pull the patient you know if we were looking for its admission or discharge summary or an HNP you know from another facility they would usually pulling that and giving it to the provider at that point." – (R5, 10y)*

Conversely, the lack of dedicated support staff posed challenges in certain settings, particularly when retrieving information that required significant attention. Several providers reported having to personally make phone calls and track down information due to limited support, especially during nights and weekends. The complexity of the process, which involved obtaining patient consent and navigating multiple hospital systems, further discouraged HIE use when support was scarce.

*"No. Um I work in a small department where the staffing is me and two nurses and sometimes a tech and usually no secretaries so there really is no nobody else to do that research for me." – (R15, 13y)*

*"Maybe 75% of the time, because then the secretary has to call to the hospital and try to find whoever can be medical records, I work nights and weekends. So on nights and weekends, a lot of medical records staff are gone. – (R2, 14y)*

#### *Theme: Training*

Some providers reported receiving initial guidance on how to log in and navigate the basic HIE system, which allowed them to begin incorporating it into their practice. The presence of more experienced colleagues who could offer informal training and support was viewed as helpful. These colleagues often became informal mentors, sharing shortcuts and tips, which motivated providers to engage with HIE systems more effectively. The role of peer support as a motivator in navigating HIE platforms is further underscored by a quote from provider: *"A colleague mentioned [the system] as being useful, so that's how I found out about it" – (R3(1y)*. A provider shared experience of navigating HIE system through "trial and error" and mentioned*, "I just sort of started using it and figured it out myself" (R11, 4y).*

However, the absence of structured and ongoing training on system capabilities and advanced features was identified as a significant barrier. Providers noted that training was often limited to basic access, without exploring the full potential of the HIE system, leaving them feeling unsupported and inefficient. Many reported that the lack of formal onboarding for the HIE system contributed to a steep learning curve. Additionally, the absence of regular updates or refresher courses on new features and best practices for HIE use was cited as a further obstacle to optimal system utilization.

*"We don’t really include it in our formal onboarding or training of physicians." - (R1, 4y)*

*"There may be more efficient ways to use it and find information that I’m just not aware of." - (R3, 1y)*

*"but now there was never like um, information session on how it can be used in a different, different ways that you can navigate through it that might be more beneficial." -(R3, 1y)*

#### *Theme: Technical support*

Providers indicated that technical issues have substantially decreased in recent years. The availability of 24/7 technical support was seen as a positive development, improving providers’ attitudes towards HIE usability.

*"I don't feel as frustrated now because I know I can rely on 24/7 technical support—there's always someone available." - (R14, 14y)*

*"3 years ago it happened all the time and now like I don't know maybe really infrequently" – (R5, 10y)*

Along with lack of comprehensive training, some providers still reported persistent technical issues. Additionally, intermittent problems, such as non-functioning links for certain users or information disappearing unexpectedly, were noted by some providers.

*"I've also noticed, sometimes the information will initially be there. And then you go back and then it's gone" - (R14, 14y)*

### Domain: Information Quality

Providers shared their insights into the usability of HIE particularly, quality of information, obtaining desired information, issues with missing information, and usability of information in emergency department.

#### Theme: Desired information

ED providers expressed a strong desire for detailed past records to avoid redundant tests and to understand the patient's medical history for making informed clinical decisions. Many providers highlighted the importance of receiving up-to-date lab results and other critical information, including admission and discharge summaries, encounter notes, and medication histories.

*“I need to make sure that they haven't had seven CT scans already in 2020.”- (R2, 14y)*

*“Mostly labs or imaging times I will look at old progress notes from a provider just to see if there's been any mention a plan, a treatment plan or a specific diagnosis that they were considering at that time on site, look at the notes as well.” - (R3, 1y)*

Respondents also discussed challenges accessing recent diagnostic tests, laboratory results and treatment is crucial for ED providers who need to make quick decisions based on the latest data. One provider emphasized.

*“I need to look and see if someone has recently had a CT scan of their belly because they’re there for belly pain.” - (R2, 14y)*

ED providers noted instances where patients physically present in the ED could not have their records accessed immediately if they had not yet been registered in the system. For example, one provider described a situation where a patient’s test results from another facility were not accessible due to delays in registration or data upload, leading to potential gaps in the patient's medical history

*“The patient has to be registered at your site to be able to access CareWeb. And sometimes there could be delays and registration. So, the patient may physically be in my emergency department. But when I log into CareWeb, the window doesn't open up until they're registered there” - (R8, 13y)*

Some ED providers prefer to review imaging reports for quick insights, while others find value in examining raw imaging data. Balancing between accessing comprehensive reports and raw data can influence the efficiency of the diagnostic process in the ED.

*“For a CAT scan, it would take a long time to go through the scan and review it all, so I would rather have the report.” - (R21, 22y)*

Similarly, providers expressed a need for the inclusion of specialist consultation notes in HIE systems which can greatly enhance the completeness of patient records. Similarly, providers expressed frustration with the absence of daily progress notes or detailed clinical notes, which are crucial for understanding patient conditions and treatment recommendations.

*A lot of specialists' notes are not always in there.” - (R19, 1y)*

*“I feel like my access to the notes was much better before. Now when I try to follow patients, I can't see daily notes. Sometimes all I can see are specialist notes and labs, which aren't always helpful.”- (R12, 2y)*

#### Theme: Missing information

One of the recurring themes among respondents was the challenge of incomplete or missing information within CareWeb hindering providers to provide comprehensive patient care. Providers expressed frustration with incomplete or missing records, which hindered their ability to deliver optimal patient care. Providers also frequently encountered delays in the uploading of crucial records, such as discharge summaries. Such delays were particularly problematic when patients moved between facilities, resulting in critical information being unavailable when needed. A provider highlighted the challenge of accessing up-to-date discharge summaries, noting:

*“I’ve had this before where a patient was recently discharged, but the actual discharge summary is not in the system yet”. - (R8, 13y)*

On the other hand, the availability of specific, timely information such as lab results or admission notes were seen as highly valuable.

*“It’s been invaluable to be able to look at their last admission, HMP, and discharge summary.” - (R12, 2y)*

The limitations of the system in handling specific types of data also emerged as a challenge. Many providers reported difficulties in accessing imaging studies and their results, as sometimes only the reports were available without the actual image. For instance, providers reported difficulties in accessing imaging studies and their results, as sometimes only the reports were available without the actual images.

*“You can see that an imaging study was done, but sometimes it's a little tricky to actually see the images instead of just a report.” - (R15, 13y)*

Another aspect of missing information involved data from specialized care settings, such as psychiatric facilities. Providers noted that information from psychiatric admissions often did not appear in the HIE system, necessitating additional efforts to track down this information through other means

*“Most patients get cardiology care at XYZ, and I almost never find an EKG from them.” - (R11, 4y)*

#### Theme: Missing institutions

Respondents reported that there is inconsistent availability of patient records from certain healthcare institutions outside the hospital network, which often hinders the seamless exchange of health information and complicates patient care. This issue is particularly frustrating when managing patients who require urgent or specialized care, as the absence of critical information can delay decision-making and lead to redundant or unnecessary procedures. Also, providers frequently encounter such situations where records were missing for patients who received care at smaller or independent facilities located outside the region. One provider expressed the frustration of managing cases without access to essential records:

*“We have a lot of XYZ kids and instead of going there, where most of the care is delivered, they come down to ABC and then we don’t have any records to be able to see what’s going on with them.”- (R2, 14y)*

*“The VA has no records online that I know of anywhere. So, you always have to call and get records from there if you are trying to look into anybody’s records from the VA.”- (R1, 23y)*

#### Theme: Usability of information

Overall, ED providers generally perceived the HIE systems, particularly CareWeb, as highly beneficial. Many providers expressed that when accessible, the information was highly beneficial, particularly in ensuring timely and informed patient care. One provider noted, “*Oh, for sure, definitely*,” when asked if the information was helpful. Another provider shared a similar sentiment, emphasizing the convenience as long as the information was readily available: “*As long as we can get to it, that’s super helpful.*”

Despite the advantages, some challenges were highlighted, particularly regarding the organization and retrieval of information. Providers often encountered difficulties in navigating the HIE systems, frequently described as time-consuming and sometimes overwhelming in navigating and extracting useful information from CareWeb. This sentiment was confirmed by another provider who pointed out the challenge of making the retrieved information clinically useful, despite being able to find it.

*“It’s organized by ID, so you kind of have to know what you’re looking for…there’s not a whole lot of extra time to be looking through chart information.” – (R3, 1y)*

*“It’s not so much finding it, it’s processing the information and making it useful…you may be able to find it, but being able to actually digest it and make it clinically useful takes a little more time.” – (R8, 13y)*

### System Quality

#### *Theme: Network reliability*

A stable network connection was reported as essential for accessing real-time patient information. Providers highlighted improvements in network infrastructure, such as the implementation of Virtual Private Network, VPN) connections for remote access, which enabled seamless access to HIE data even from home. One provider noted that these enhanced network solutions reduced technical barriers, thus motivating increased HIE use.

*“...hospitals created that VPN connection, so I don’t have a problem with that anymore,” (R8, 13y)*

*“...I can pull up the CareWeb, but if I’m just on my regular home browser, that doesn’t work.” - (R8, 13y)*

Interruptions caused by regular system upgrades, often occurring during critical night shifts or weekends, were commonly cited as demotivating factors by providers. Network outages, whether due to internet failure or slow system performance, also contributed to this challenge. Another provider described these issues as a significant barrier, particularly during peak work hours.

*“We have shut down to where they do upgrades...probably once or twice a month,” which disrupted their ability to retrieve patient data promptly. - (R2, 14y)*

*“...if it’s running slow for some reason...if there’s server issues or if it’s on our end, the network connectivity, that would be [a challenge].” - (R21, 22y)*

#### *Theme: System reliability*

Improvements in system functionality contributed to a more seamless experience, with features such as automatic sign-on integration between systems like CareWeb and Epic being especially appreciated. The consistent availability of HIE data, particularly following system upgrades, also enhanced providers' confidence in relying on the technology to streamline their clinical operations.

*“...if that link works...it’s so much more useful and quicker.” – (R1, 23y)*

*“The integration with Cerner and just a more reliable system have both helped.” – (R15, 13y)*

On the other hand, dissatisfaction with system reliability persisted, particularly regarding downtime during night and weekend shifts when technical support was less accessible. Recurring issues such as system slowness, login failures, and overall performance degradation interrupted workflow, often falling short of providers' expectations.

*“Every time I try to use the single sign-on...it doesn’t work for some reason.” – (R11, 4y)*

*“...if the system's down and I can't, I can't find it.” – (R8, 13y)*
