# Supplementary material for: Influences on Emergency Clinician Use of Health Information Exchange: Interview Study
Source: JMIR Med Inform. 2025 Oct 20;13:e75865. doi: 10.2196/75865 (PMC12583940; doi:10.2196/75865)
Supplement: Multimedia Appendix 2 [file medinform_v13i1e75865_app2.docx]

# Multimedia Appendix 2

Influences on Emergency Clinician Use of Health Information Exchange: Interview Study from the Indiana Network for Patient Care

doi: 10.2196/75865

# Interview Guide

**Introduction**

Thank you for agreeing to speak with me; I appreciate your time.

This interview is focused on your access to patient information that is generated by another hospital, clinic or health system. We are interested in why, when, and how you access “outside” or “external” information you need to take care patients. Your input will help us design tools and methods for improving access and efficiency with respect to outside patient information.

This interview will take approximately 40 minutes.

If you agree, I'd like to record this interview; the audio tape will be used to help with note-taking. The information you share will not be connected to your name in any reports, publications or presentations of the research results. All information from this interview will be confidential. Your name will be known only to the study team. If names come up during the interview they will be removed from my notes and the audio recording to maintain your anonymity and the confidentiality of all information you share. If, at any time, you wish to stop the interview please let me know and I will end turn off the recorder.

Do you consent to being interviewed? *If participant agrees, recorder is turned on*.

Just a reminder that the audio recorder is now on. Is that ok?

Thank you for agreeing to be interviewed.

First, a few questions about your background and position, and about your experiences accessing outside information on a patient.

**1. Background**

Let’s start with a little background information.

1A. What is your title? [*check whether full- or part-time*]

1B. How long have you been a [DOCTOR, NURSE, MA, OR OTHER]?

1C. How long have you worked at NAME OF HOSPITAL OR CLINIC?

1D. Describe your role within the [UNIT OR TEAM]?

1E. What percentage of your time do you spend seeing patients (or working clinically)?
[e.g., as opposed to administrative work or research duties]

**2. Your Work and Looking for Information from Outside Providers**

We recognize that you spend time looking within your EHR system to find information about a patient who comes to your [UNIT OR TEAM] for care. While there is likely some information on the patient if they visited your hospital in the past, it is equally as likely that there is information on this patient from care they received at [NAME OF COMPETING OR OTHER HOSPITAL]. We are interested in how and when as well as why you access information from so called “outside providers” in the course of treating a patient.

2A. Describe a **common scenario** where you would need to look for or request information from outside providers?

Probes: How do you know that you need to look for or request information from outside?

2B. Describe **how** you typically search for information from outside providers.

Probes: Do you use your EHR? Do you use another information system? What is the name of the system you use? Do you call outside provider offices? Does someone do this work for you?

[If respondent does not use any methods for searching] Why do you not seek information from outside providers?

2C. How often are you able to successfully obtain the information from outside providers?

Probes: In what form do you get the information? (phone call, discrete, faxed, searchable, etc); Is there some characteristic about the patient that allows you to get the information in a certain format (geography, technology, type of patient)?

2D. Describe where in the care delivery process you would typically search for information from outside providers?

Probes: Is there a critical point in time when outside information would be useful?

2E. Can you estimate the proportion of time you spend gathering (or tracking down) information from outside providers?

**3. Perceptions Related to INPC CareWeb**

Your hospital has access to information from outside providers via an information system entitled CareWeb from the Indiana Health Information Exchange. The CareWeb application can be accessed via [YOUR EHR SYSTEM OR AN INTERNET BROWSER]. Once you have logged into CareWeb, you have access to browse or search for information from outside providers. We would now like to ask you question about CareWeb and your usage of CareWeb.

3A. Describe your knowledge and use of the CareWeb application.

Probes: Do you use it? When was the last time you logged into CareWeb? How do you access it from your EHR? How often do you use it in a week? Do you log in multiple times during a patient encounter?

3B. What do you believe are the **advantages** of accessing the INPC CareWeb system to retrieve outside information such as encounters, laboratory values, imaging studies, or physician notes?

Probes: Do you think the information in CareWeb improves the quality of care you provide?

3C. What do you believe are the **disadvantages** of accessing the INPC CareWeb system to retrieve prior information such as encounters, laboratory values, imaging studies, or physician notes?

Probes: Do you think the information from CareWeb negatively impacts care quality?

3D. Is there anything else you associate with your own views about accessing the INPC CareWeb system to retrieve outside information such as encounters, laboratory values, imaging studies, or physician notes?

Probes: Can you describe a time when you used CareWeb and it changed your decision-making? Can you describe when using CareWeb might have made a difference in the care you delivered?

3E. Are there any colleagues who would **approve** of your accessing the INPC CareWeb system to retrieve outside information such as encounters, laboratory values, imaging studies, or physician notes?

Probes: Which types of colleagues approve?

3F. Are there any colleagues who would **disapprove** of your accessing the INPC CareWeb system to retrieve outside information such as encounters, laboratory values, imaging studies, or physician notes?

Probes: Which types of colleagues disapprove?

3G. Is there anything else you associate with other people’s views about accessing the INPC CareWeb system to retrieve outside information such as encounters, laboratory values, imaging studies, or physician notes?

3H. What factors or circumstances would **enable** you to access the INPC CareWeb system to retrieve outside information such as encounters, laboratory values, imaging studies, or physician notes?

Probes: What about a button from within the EHR? What if the information were integrated into the EHR? How could your workflow change to enable access to CareWeb? Are there any organizational policies or procedures that might enable access to CareWeb?

3I. What factors or circumstances would **make it difficult or impossible** for you to access the INPC CareWeb system to retrieve outside information such as encounters, laboratory values, imaging studies, or physician notes?

Probes: Would any modifications to your workflow make it hard to access CareWeb? What kinds of rules or organizational policies might make it difficult to access CareWeb? Are there any EHR system changes that would make accessing CareWeb hard?

3J. Are you aware that CareWeb contains information from the VA on Veterans who receive much of their care within the VA system?

Probes: Do you ever use CareWeb to look up information on Veterans?

3K. Are you aware that the Indiana HIE monitors ED visits, sending electronic notifications to payors, ACOs, and other health systems where the patient receives their primary care?

Probes: How do you feel about this? Has anyone from a payor or the VA ever contacted you about an ED visit for which they received an electronic alert?

3L. Are there any other issues that come to mind when you think about access the INPC CareWeb system to retrieve outside information such as encounters, laboratory values, imaging studies, or physician notes?

Probes: Can you tell me about a time when you really needed CareWeb to work or needed something, but it wasn’t there?

**4. FINAL QUESTIONS for ALL Respondents**

4A. What else about your work, accessing outside information, or CareWeb that hasn’t come up yet that you think would be good for the study team to know?

Thank you again for your time.
